# Bioadhesive 3D-Printed Skin Drug Delivery Polymeric Films: From the Drug Loading in Mesoporous Silica to the Manufacturing Process

**DOI:** 10.3390/pharmaceutics15010020

**Published:** 2022-12-21

**Authors:** Rafaela Santos de Oliveira, Nadine Lysyk Funk, Juliana dos Santos, Thayse Viana de Oliveira, Edilene Gadelha de Oliveira, Cesar Liberato Petzhold, Tania Maria Haas Costa, Edilson Valmir Benvenutti, Monique Deon, Ruy Carlos Ruver Beck

**Affiliations:** 1 Programa de Pós-Graduação em Ciências Farmacêuticas, Faculdade de Farmácia, Universidade Federal do Rio Grande do Sul, Porto Alegre, RS 90610-900, Brazil; 2 Laboratório de Nanocarreadores e Impressão 3D em Tecnologia Farmacêutica (Nano3D), Faculdade de Farmácia, Universidade Federal do Rio Grande do Sul, Porto Alegre, RS 90610-000, Brazil; 3 Instituto de Química, Universidade Federal do Rio Grande do Sul, Porto Alegre, RS 90650-001, Brazil; 4 Departamento de Farmacociências, Universidade Federal de Ciências da Saúde de Porto Alegre, Porto Alegre, RS 90050-170, Brazil

**Keywords:** 3D printing, clobetasol propionate, drug, nanomaterial, semisolid extrusion, mesoporous silica

## Abstract

The alliance between 3D printing and nanomaterials brings versatile properties to pharmaceuticals, but few studies have explored this approach in the development of skin delivery formulations. In this study, clobetasol propionate (CP) was loaded (about 25% *w/w*) in mesoporous silica nanomaterial (MSN) to formulate novel bioadhesive and hydrophilic skin delivery films composed of pectin (5% *w/v*) and carboxymethylcellulose (5% *w/v*) by 3D printing. As a hydrophobic model drug, CP was encapsulated in MSN at a 3:1 (*w/w*) ratio, resulting in a decrease of CP crystallinity and an increase of its dissolution efficiency after 72 h (65.70 ± 6.52%) as compared to CP dispersion (40.79 ± 4.75%), explained by its partial change to an amorphous form. The CP-loaded MSN was incorporated in an innovative hydrophilic 3D-printable ink composed of carboxymethylcellulose and pectin (1:1, *w/w*), which showed high tensile strength (3.613 ± 0.38 N, a homogenous drug dose (0.48 ± 0.032 mg/g per film) and complete CP release after 10 h. Moreover, the presence of pectin in the ink increased the skin adhesion of the films (work of adhesion of 782 ± 105 mN·mm). Therefore, the alliance between MSN and the novel printable ink composed of carboxymethylcellulose and pectin represents a new platform for the production of 3D-printed bioadhesive films, opening a new era in the development of skin delivery systems.

## 1. Introduction

Mesoporous silica nanomaterials (MSN) are inorganic-based nanostructures that have been widely applied as nanocarriers over the last two decades, starting from the first report of MSN-based drug delivery system for ibuprofen in 2001 [1]. The current inexhaustible interest of researchers in these nanomaterials is mainly thanks to the presence of mesopores, which lead to a high surface area and large pore volume; both excellent features to achieve a high drug-loading capacity. Moreover, their highly ordered, uniform and tuneable pores (typically 2–6 nm in diameter) afford a fine control of drug loading and increased drug release kinetic profiles [2,3]. The versatile characteristics of silica are also acquired by MSNs, such as a rigid and thermally stable framework, easy surface modification and good biocompatibility, and are recognised as safe systems by the United States Food and Drug Administration (FDA) [4]. The combination of these characteristics makes MSNs ideal platforms to transport and protect drugs efficiently in the development of nanomedicines [5,6].

Among the different types of MSNs, MCM-41 is well-known for its high surface area (900–2100 m²/g), pore dimensions between 1.5 and 8 nm, pore volume higher than 1 cm³/g and 2 D hexagonal porous structure [7]. These properties make them promising candidates for the delivery of poorly water-soluble drugs, showing great potential in the delivery of drugs to the skin [8]. The loading of a drug on MSNs strongly depends on the adsorptive properties of the MSN, as the drug–carrier interactions must be protagonists to minimise the fraction of the drug adsorbed on the outer particle surface so as to avoid its crystallisation due to the absence of (pore) confinement effects [9,10]. An amorphous state of the drug is desired, especially for poorly soluble drugs, as the conversion of the crystalline state to the amorphous form can improve the drug dissolution/release rate and bioavailability [11,12]. In this regard, the low aqueous solubility of some active pharmaceutical ingredients for skin treatments makes their formulation in aqueous delivery systems difficult, resulting in some penetration/permeation drawbacks, which consequently influence further clinical effects [13]. Therefore, the encapsulation of lipophilic drugs in MSNs has been highlighted as a very interesting strategy, because they hold outstanding characteristics for controlling the drug dissolution profile and can be used to develop solvent-free and oil-free skin delivery formulations. In addition, as previously reported, MSNs can improve drug accumulation and retention in the skin, which are desired properties for drugs that act locally in the skin or mucosa [8,14,15,16].

However, it is noteworthy to plan how these nanomaterials would be applied to the skin and how could they be personalised for specific dermatological disorders and patients. With these purposes in mind, 3D printing has been reported as a strategy for the preparation of topical drug dosage forms, as it brings irrefutable benefits for the patient, allowing customisation of the size, shape and swelling profile, and modulation of drug release, pore structure and disintegration time, among others, which are fundamental features for an effective and comfortable skin therapy [17]. Moreover, 3D printing also enables a reproducible solvent-free process that can not be achieved by conventional methods such as solvent casting and electrospinning, which have poor shape reproducibility and can result in organic solvent residues in the final dosage form (Ghofrani et al. 2022). Given this reasoning, our group has been exploring the alliance between drug-loaded nanomaterials and 3D printing, to improve the physicochemical and biopharmaceutical properties of such 3D-printed dosage forms [18] as oral [19,20] and buccal drug delivery systems [21]. However, to the best of our knowledge, there is a lack of studies proposing the use of the alliance between MSNs and 3D printing as a suitable approach to formulate mucoadhesive and hydrophilic 3D-printed polymeric films for skin delivery.

Semisolid extrusion (SSE) has been gaining popularity as a 3D printing technique in pharmaceutics in the last years, due to its versatility, the possibility of using it at low temperatures, the blending of different polymers to provide specific features to the formulation, and the easy modulation of the semisolids’ properties, among others [18,20]. Carboxymethylcellulose (CMC), a well-known polymer with good rheological properties, was previously reported by our group as a suitable polymer to prepare printable inks containing organic nanomaterials [20]. However, in the development of skin delivery films, the bioadhesion properties must be enhanced. Pectin, a polymer widely used in commercial dressings, which shows good bioadhesion properties [17], may be suggested as a component of such films, able to promote a cutaneous therapy with good performance and aesthetic acceptance.

Therefore, this study was designed to load a drug in MSNs for the formulation of 3D-printed bioadhesive films, as novel skin delivery systems. Clobetasol propionate (CP), a glucocorticoid drug, was chosen as the model drug, due to its lipophilic properties and its well-known dermatological use. The use of a hydrogel composed of CMC and pectin was evaluated as a printable ink in the preparation of 3D-printed bioadhesive films using the SSE technique. In addition, the effect of the presence of pectin on the bioadhesion of the films was evaluated using porcine skin. To the best of our knowledge, this is the first report proposing this polymeric blend to produce skin delivery systems by 3D printing.

## 2. Materials and Methods

### 2.1. Materials

Clobetasol propionate, carboxymethylcellulose (FH 4000) and pectin were acquired from Levviale (Anápolis, Goiás, Brazil). The MCM-41 type (hexagonal) MSN was purchased from Sigma-Aldrich (São Paulo, São Paulo, Brazil). Ethanol analytical grade was obtained by Neon (Suzano, São Paulo, Brazil). All reagents and solvents were analytical or HPLC grade and were used as received.

### 2.2. Drug Loading

CP was incorporated into the MSN using the solvent evaporation method [9]. MCM-41 powder was added to an ethanolic CP solution (6 mg mL^−1^) at a MCM-41:CP ratio of 3:1 (*w/w*). This dispersion was kept under stirring for 24 h at 37 °C. The solvent was then evaporated under reduced pressure in a rotary evaporator at 40 °C. Then, the formulation was further dried at 50 °C to a constant weight. The resulting CP-loaded MSN material was labelled as MSN-CP-3-1. No further drying was performed, unless stated in the following characterisation sections. The process yield (%) was calculated by the ratio between the final weight of the powder recovered at the end of the drug-loading process and the total solid mass added.

### 2.3. Drug Content

CP was assayed by high performance liquid chromatography (HPLC) according to a method previously reported [22], with slight modifications. The system consisted of a Shimadzu LC-System (Kyoto, Japan) equipped with a LC-20 AT pump, SPD-20 AV detector (UV detector), DGU-20 A5 degasser, CBM-20 A system controller, SIL-20 A auto sampler and LC-Solution, and a Gemini column (C18, 150 mm × 4.6 mm), with the following parameters: injection volume of 20 µL, 1 mL min^−1^ isocratic flow rate and wavelength detection at 241 nm. The mobile phase was composed of methanol-water (80:20 *v/v*). For the drug extraction for the drug content analysis, approximately 10.5 mg of each formulation were weighed and placed in 5 mL volumetric flasks filled with ethanol. The mixture was kept under moderate stirring for 30 min at 37 °C, sonicated for 40 min and centrifuged at 2325 g for 15 min. The supernatant was diluted in mobile phase and analysed by HPLC. The drug loading was calculated using Equation (1).

(1) $$\mathrm{Drug}\;\mathrm{loading}\;(\%)=\frac{\mathrm{Experimental}\;\mathrm{drug}\;\mathrm{content}\;(\mathrm{mg})}{\mathrm{Total}\;\mathrm{sample}\;\mathrm{weight}\;(\mathrm{mg})}$$

### 2.4. Scanning Electron Microscopy (SEM)

The morphology of the MSN was evaluated in a scanning electron microscope (EVO MA10, Zeiss, Oberkochen, Germany). Before analysis, the samples were dispersed on double sided conductive tape on an aluminium support.

### 2.5. Thermogravimetric Analysis (TGA)

TGA was performed using a thermoanalyser (Shimadzu Instrument model TGA-50, Kyoto, Japan), with a heating rate of 20 °C min^−1^ in a 50 mL min^−1^ nitrogen flow, from room temperature up to 900 °C.

### 2.6. Nitrogen Adsorption-Desorption Isotherms

Surface area and pore volume characterisation was performed using N_2_ adsorption-desorption isotherms at liquid N_2_ boiling point temperature, using a surface area and porosity analyser (Tristar II Kr 3020, Micromeritics, Norcross, GA, USA). The samples were previously degassed at 60 °C for 12 h, under vacuum. The specific surface area was determined by the BET (Brunauer, Emmett and Teller) multipoint technique, and the pore size distribution was obtained using the BJH (Barret, Joyner and Halenda) and DFT (Density Functional Theory) methods (Webb, Orr and Camp, 1997).

### 2.7. Differential Scanning Calorimetry (DSC)

DSC analyses were performed using a differential scanning calorimeter (Shimadzu DSC-60, Kyoto, Japan). The 1–2 mg samples were weighed in aluminium pans, and the analyses were carried out at a heating rate of 10 °C min^−1^, from 30 °C to 250 °C. Nitrogen gas was used at a flow rate of 50 mL min^−1^. The following samples were analysed: crystalline CP (raw material), MCM-41 raw material, MSN-CP-3-1, and its respective silica:drug physical mixture (PM-MSN-CP-3-1).

### 2.8. X-ray Powder Diffraction (XRD)

The XRD patterns were evaluated using a diffractometer (Ultima IV, Rigaku, Tokyo, Japan), with CuKα radiation (λ = 0.1542 nm) at 40 kV and 17 mA. The following samples were analysed: MCM-41 raw material, CP raw material and MSN-CP-3-1.

### 2.9. In Vitro Drug Dissolution Studies

In vitro drug dissolution studies were carried out by the dialysis bag method (molecular weight cut off 14,000 kDa, Sigma-Aldrich, São Paulo, São Paulo, Brazil), using a mixture of 70:30 *v/v* phosphate buffer solution 0.1 mol L^−1^ (PBS 0.1):ethanol as the release medium [23,24]. MSN-CP-3-1 samples (*n* = 3) equivalent to 0.5 mg of CP were weighed, placed in the dialysis bag with 1.0 mL of the release medium, and subsequently immersed into 200 mL medium. Aliquots (1.0 mL) were collected at predetermined times (0.25, 0.5, 1, 2, 2.5, 3, 4, 5, 6, 8, 12, 24, 48 and 72 h) and immediately replaced with 1.0 mL of fresh medium. The experiment was kept at 37 °C and constantly stirred. Ethanolic CP solution and crystalline CP (raw material) dispersed in the release medium (as a suspension) were evaluated as controls. CP was assayed in the collected samples by HPLC, according to the method previously described (Section 2.3); however, the injection volume was increased to 100 µL to lower the limit of quantification. The chromatographic method was linear (r = 0.9989; *n* = 3) in the range between 0.25 and 5 µg mL^−1^ and was considered specific for CP assay, as no interference of the medium components was detected. Data from the in vitro drug release studies were plotted as the cumulative percentage of drug release versus time. The dissolution efficiency (DE) was calculated from the dissolution profiles shown by each sample, using the trapezoidal method and considering the area under the curve (AUC) over the 72 h of the experiment [25].

### 2.10. Preparation of the 3D Printing Inks

Hydrogels were prepared as printing inks by separately dispersing CMC (5% *w/v*) and pectin (5% *w/v*) in distilled water at 65 °C. Glycerine (15% *w/v*) was incorporated in the CMC hydrogel until a smooth hydrogel was formed. Once dispersed, the CMC and pectin hydrogels were blended in a 1:1 (*w/w*) ratio until a homogenous gel was obtained. The final composition of the printing ink was CMC 2.5% (*w/v*), pectin 2.5% (*w/v*) and glycerine 7.5% (*w/v*). MSN-CP-3-1 was then added to the hydrogel, followed by a careful homogenisation step, to obtain a final concentration of ca. 0.09 mg/g of CP (HG-MSN-CP). After preparation, the printing ink was immediately packed into 3D printing syringes and stored in the refrigerator until use. A hydrogel without MSN-CP-3-1 was prepared for comparison (HG).

### 2.11. Rheological Characterisation of the 3D Printing Inks

Texture analyses of the hydrogels were carried out using a texture analyser (TA.XT plus, Stable Micro Systems, Godalming, Waverley, UK), using two techniques: back extrusion and forward extrusion. For the backward extrusion test, a stainless-steel cylindrical probe with a spherical plastic tip (35 mm) was used to penetrate 25.0 mm into the gel at a rate of 2.0 mm/s. The disc compression extruded the product up and around the edge of the disc. The result was digitally recorded. For the forward extrusion test, a piston disc was used to compress the sample by 20.0 mm at a speed of 1.0 mm/sec through a base disc with an outlet diameter of 3 mm, leading to forward flow. The force required to enable the piston disc to extrude the sample through the standard hole was digitally recorded.

Rheological analyses were performed using an Ares G2 rheometer (TA Instruments, New Castle, DE, USA) with a parallel plate geometry (25 mm diameter and measuring gap of 1 mm) at 25 °C. The linear viscoelastic region (LVR) and estimated yield stress value (Pa) were determined by an oscillation amplitude test (1.0 to 500% strain sweep and 1 Hz frequency). The viscoelastic properties of the hydrogels were measured by an oscillation frequency test at a constant strain of 0.1% and an angular frequency sweep of 0.1 to 400 rad/s. Apparent viscosity, storage modulus (G’), loss modulus (G’’) and tan δ (G’’/G’) were recorded. A flow ramp test, with a shear rate range of 1.0 to 5.5 s^−1^, was conducted to determine the best-fit flow. Data collection and analyses were performed using Trios software (v.4.4.0, TA Instruments, New Castle, DE, USA), and all rheological measurements were carried out in triplicate.

### 2.12. 3D Printing of the Skin Delivery Films

The external geometry of the 3D-printed films was designed to be 15 mm (length) × 15 mm (width) × 1 mm (height) using the Prusa Slicer software (Prusa Research, Prague, Czech Republic) and exported as a stl file. The same software was used to slice the 3D geometric design into G-code to communicate with the 3D printer. The CMC-pectin films were printed using a semisolid 3D printer (3DBioEdPrinter V2, BioEdTech, São Paulo, Brazil). The printing process was carried out at room temperature, and the following parameters and conditions were set, according to preliminary tests: extrusion speed 6 mm·s^−1^, filling percentage 100%, layer height 0.2 mm, nozzle diameter 0.41 mm, and dimensions 15 mm × 15 mm × 1 mm. Each film had a printing time of approximately 7 min. After printing, the films were left at 25 °C for 72 h for water evaporation. The 3D-printed films were labelled as F-HG-MSN-CP and F-HG for films prepared from HG-MSN-CP and HG inks, respectively.

### 2.13. Physicochemical Characterisation of 3D-Printed Films

The mean weight of the films was evaluated using a calibrated analytical balance after drying for 72 h (*n* = 10). The mean thickness and dimensions were measured with a digital calliper (Digimess, São Paulo, São Paulo, Brazil) (*n* = 3). The CP content of the 3D-printed films (*n* = 3) was assayed by placing each film in a 10 mL volumetric flask containing 2 mL purified water, followed by 20 min magnetic stirring and 15 min in an ultrasonic bath (*n* = 3). Then, approximately 5 mL of methanol was added, followed by 15 min magnetic stirring and 100 min in an ultrasonic bath. The samples were centrifuged, filtered and analysed by HPLC, according to the method previously described (Section 2.3).

### 2.14. Mechanical Properties of 3D-Printed Films

The tensile strength of the 3D-printed films (*n* = 3) was measured using a texture analyser (TA.XT plus, Stable Micro Systems, Godalming, Waverley, UK) equipped with a 5 kg load cell and the apparatus tensile grips. For the experiment, special size films were printed (55 mm × 10 mm × 1 mm) to fit the requirements of the apparatus and were vertically clamped between two tensile grips. The initial gauge length was set to 31 mm, and each film was stretched at a crosshead speed of 0.5 mm·s^−1^ until it broke. During stretching, the elongation at break (mm) and the respective force (*n*) were recorded. Texture Exponent software (Stable Micro Systems, Godalming, Waverley, UK) was used for data collection and to calculate the force, distance, stress and strain. All tests were carried out in triplicate.

### 2.15. In Vitro Swelling Properties of 3D-Printed Films

The swelling properties of the 3D-printed films were determined using a gravimetric method. The film samples were accurately weighed and placed on a petri dish containing 7 mL phosphate buffer 0.1 mol L^−1^ pH 7.4. At predetermined time intervals, the film was withdrawn and the excess PBS was gently removed from the surface with a tissue paper. The samples were immediately weighed to determine the wet weight. The weight of each sample was monitored until there was a loss of the film integrity.

The swelling index was calculated using Equation (2) (*n* = 3):

(2) $$\mathrm{Swelling}\;(\%)=\frac{\left(Ww-Wd\right)}{Wd}\;\times \;100$$

where Ww is the weight of wet film at a particular time (*t*) and Wd is the weight of the dry film (before swelling).

### 2.16. In Vitro Drug Release from 3D-Printed Films

In vitro drug release studies were performed using Franz diffusion cell, with a diffusion area of approximately 2.096 cm² and receptor compartment of approximately 6.6 mL. A dialysis cellulose membrane (molecular weight cut off 14,000 kDa, Sigma-Aldrich, São Paulo, São Paulo, Brazil) was placed between both compartments. The receptor medium was composed of PBS 0.1:ethanol (70:30 *v/v*) to ensure sink conditions during the experiment. The films (F-HG-MSN-CP) were applied to the dialysis cellulose membrane (*n* = 3), and the receptor medium was stirred during all experiments and maintained at 37 °C. Aliquots of 150 μL were collected from the receptor medium at 0.5, 1, 2, 4, 6, 8 and 10 h, and immediately replaced with fresh medium at 37 °C. The drug content was assayed by HPLC using the method previously described (Section 2.3).

### 2.17. In Vitro Skin Adhesion Behaviour of the 3D-Printed Films

The bioadhesion experiment was carried out using a tensile stress tester (TA.XT plus, Stable Micro System, Godalming, Waverley, UK) for the 3D-printed films (*n* = 3). Porcine ears were obtained from a local slaughterhouse (Ouro do Sul, Harmonia, Brazil) and their skin used as substrate. Porcine ear samples were cleaned by removing the hair and adipose tissue. They were stored at −20 °C in aluminium foil and used within 3 months. On the day of the experiments, skin tissue was maintained under room conditions for at least 30 min prior to the experiments. A fixed volume (20 μL) of ultrapure water was pipetted onto the tissue to standardise hydration, and excess water was removed with absorbent paper. Skin pieces were fixed to the equipment probe with double-sided adhesive tape, whereas the formulations were fixed on the equipment’s platform with the aid of an instant adhesive. The equipment promoted the contact of the skin piece with the film for 3 min with a force of 290 mN. The probe with the skin was then removed from the surface of the film at a constant speed of 0.10 mm·s^−1^ until total displacement [26]. The debonding distance (mm) and the work (mN·mm) necessary to detach the skin (peaks of force (mN) x displacement (mm)) from the 3D-printed films were calculated by the software (Exponent, Stable Micro Systems, Godalming, Waverley, UK).

### 2.18. Statistical Analyses

One-way analysis of variance (ANOVA) followed by Tukey’s test (*p* ≤ 0.05) was employed for comparison of most of the experimental data. Two-way ANOVA followed by the Bonferroni test (*p* ≤ 0.05) was employed for comparison of the swelling test data. Student’s t-test was used to compare the performance of the formulations in the texture analyses (*p* ≤ 0.05). All formulations were prepared and analysed in triplicate, and values are presented as mean ± SD.

## 3. Results and Discussion

In this study, the encapsulation of a lipophilic model drug (CP) in MSN was proposed to formulate innovative 3D-printed bioadhesive skin drug delivery systems, considering that MSNs have been recently highlighted as promising platforms in the formulation of skin delivery dosage forms [27]. In the first stage, MSN-CP was synthesised at 3:1 (*w/w*) MSN:CP ratio and evaluated regarding its potential to afford partial or complete drug amorphisation. This nanomaterial was characterised regarding its physicochemical properties and in vitro drug dissolution profile. The CP-loaded MSN was then formulated into 3D bioadhesive films intended for the treatment of skin disorders. A novel polymeric blend composed of CMC and pectin was evaluated for the preparation of 3D-printed films by the SSE technique. The influence of adding pectin to the CMC hydrogel was also assessed with regard to the in vitro skin bioadhesion behaviour.

### 3.1. Physicochemical and Morphological Characterisation of Bare and CP-Loaded MSN

The SEM images of MSNs before drug loading (bare MSN) are presented as Supplementary Materials (Figure S1) and reveal agglomerates of rugous spherical and spheroidal particles with broad particle size distribution in the submicrometric range. These morphologies are in good agreement with studies performed by Hassan et al. (2015) and Shariatinia and Zahraee (2017), which also reported similar MCM-41 morphologies [28,29]. Regarding its particle size, the submicrometric range is far below the opening of the needle later used for 3D printing of hydrogel HG-MSN-CP; therefore, no blocking is expected during the extrusion process. Bare MSN was also evaluated by XRD analysis in the range of 2 tetha = 1 to 10°, showing the characteristic peaks of its hexagonal array of pores, presented by a narrow high intensity reflection (100) and by two low intensity reflections (110 and 200) (Supplementary Materials, Figure S2) [30].

CP-MSN was prepared at a silica-to-drug ratio of 3:1 (*w/w*), which corresponds to a theoretical drug loading of 0.25 mg/mg. The process yield was 66 ± 5%. This yield agrees with other drying methods, such as spray-drying [31,32], as some mass losses can occur through the process and during the handling of small quantities of powder (batch size: 240 mg).

**Figure 1:**
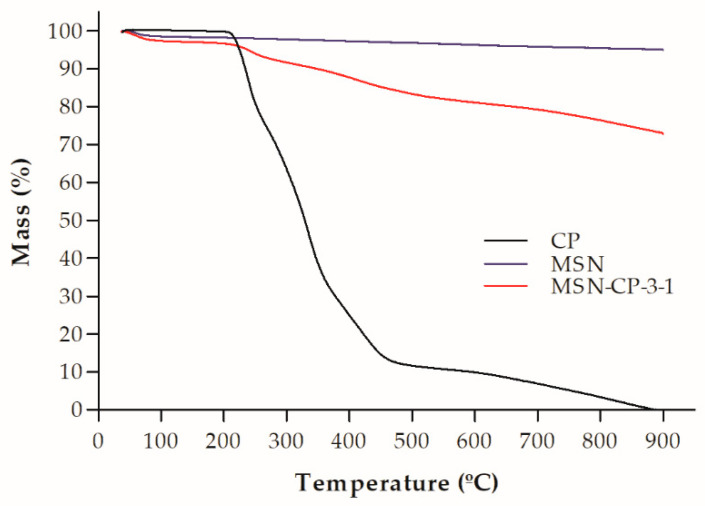
Thermogravimetric analysis of crystalline CP, bare MSN (MCM-41) and MSN-CP-3-1.

The drug content of MSN-CP-3-1 was assessed by HPLC, revealing a content of 0.23 ± 0.02 mg/mg, which agreed with the theoretical content (0.25 mg/mg), showing that there was no substantial drug loss in the loading process (>90% of drug recovery). This drug content represents a high drug loading value of about 25% (*w/w*), which could be explained by the introduction of a vacuum-drying step during the process, to remove air from the internal pores and therefore enhance drug loading in the MSNs [33,34]. The drug loading of the samples and their thermal stability were also evaluated by TGA. The thermograms are shown in Figure 1. The first weight loss of MSN-CP-3-1, from 0–150 °C, is attributed to water desorption [35]. The second weight loss region is observed between 250–450 °C, mainly attributed to organic decomposition of CP, which smoothly extends up to ca. 900 °C, with the decomposition of the remaining organic structured moieties. In the same temperature range, dehydroxylation and condensation reactions of the silanol groups from the silica surface take place, whereas silica sintering occurs above 1000 °C [36]. A clear increase of the weight loss from bare MSNs to MSN-CP-3-1 confirmed the loading of CP. Moreover, the calculated drug content of MSN-CP-3-1 from the TGA data was 0.21 mg/mg, which agreed with the experimental data obtained by HPLC analysis. These results highlight the accuracy of the methods and the data reliability.

**Figure 2:**
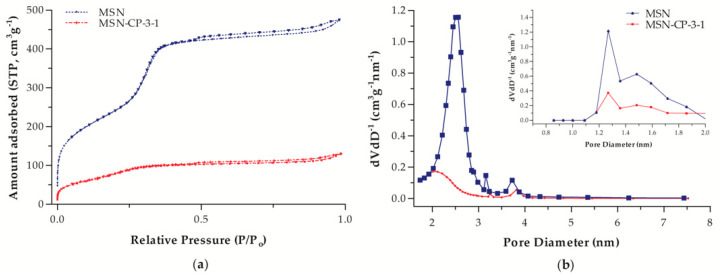
(**a**) Nitrogen adsorption and desorption isotherms and (**b**) pore size distribution of bare MSN (MCM-41) and MSN-CP-3-1.

To assess whether CP was loaded in the inner or outer surface of the MSN structure, the surface area and pore volume of the samples were evaluated by N2-adsorption-desorption isotherms, as shown in Figure 2. These data are presented in Table 1 and emphasise the inclusion of CP in the pores. The isotherms of the MCM-41 samples had a highly inflected curve regions, indicating uniform and cylindrical pores (Figure 2a). This property was confirmed by the sharp and unimodal BJH pore size distribution observed for MSNs, with maximum distribution centred at 2.5 nm, as presented in Figure 2b. For CP-MSN-3-1, the absorbed nitrogen volume in the isotherms (Figure 2a) are much lower than that for the bare MSNs, especially in the filling step of the isotherm at P/P0 < 0.3. This can be explained by the pore being filled with the CP molecules packed inside the channels.

However, the CP molecules do not fully occupy the pores, as there is still some available mesopore volume in the sample. Additionally, the pore size distribution centred at ca. 2.5 nm did not solely shift to lower values with the incorporation of CP, but also decreased greatly in volume (Figure 2b), indicating that CP is not adsorbed only as mono- or multilayers at this silica:drug ratio, but that it is indeed filling the pore [12].

**Figure 3:**
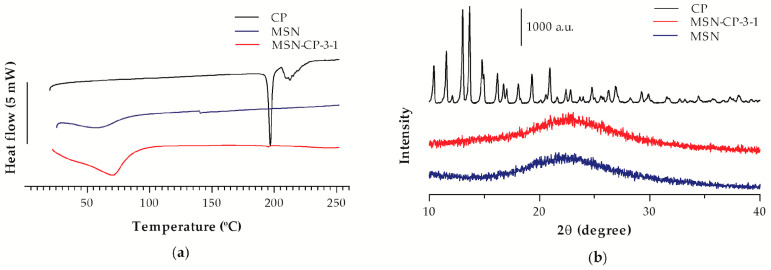
(**a**) Differential scanning calorimetry (DSC) thermal profile and (**b**) X-ray diffraction patterns of crystalline CP (raw material), bare MSN (MCM-41) and MSN-CP-3-1.

Beyond mesopores, MCM-41 presents micropores (d < 2 nm) in its surface. At low relative pressures, with P/P0 below 0.1 (micropore region), a clear decrease of the amount of adsorbed nitrogen can be observed in the CP-loaded MSN, which indicates the closure of the micropores. This feature is better observed in the DFT pore distribution (inset Figure 3b), where the micropores have a significantly lower volume for CP-loaded MSN. Considering that micropores are entirely distributed through the silica surface, their covering indicates that CP is present in most of the channels, not only on the outer surface. This hypothesis is endorsed by the surface area and pore volume values.

Thus, the presence of the CP in MSN-CP-3-1 evidenced by HPLC and TGA analysis, as well as the significant pore closure in both mesopores (pores between 2 and 50 nm) and micropores (pores smaller than 2 nm) of MSN observed by sorption analysis after CP incorporation proves the successful loading of CP into MSN. Both types of pores, micro and mesopores, are partially filled in the sample MSN-CP-3-1, suggesting drug disposition in the pore channels. This filling profile might influence the drug dissolution rate, as drug release is controlled by the diffusion of water molecules into the pores, among other mechanism. Therefore, completely filled pores would increase the drug-drug interactions and hinder water diffusion, especially when filled with hydrophobic drugs such as CP, thus slowing the release rate. In this regard, MSN-CP-3-1 is expected to present an initial burst release due to the higher water diffusion into the pores [37]. However, solid state studies must be carried out in parallel to the surface area and pore volume analyses to assess the degree of crystallisation of the drug. In general, to stay in the amorphous form, the amount of drug should be small and highly dispersed in the silica material. If overloading occurs, the layer of drug exceeds the critical thickness and crystallisation might occur [10]. Thus, DSC and X-ray analysis were carried out to further understand the structural organisation of the developed CP-MSN-3-1 formulation and to predict its drug dissolution behaviour.

The DSC thermal profiles are shown in Figure 3a. The crystalline CP thermal profile is characterised by an intense endothermic peak at 195 °C, while bare MSN did not display any peak. A weak endothermic peak was observed in the thermogram of MSN-CP-3-1, indicating the presence of traces of crystalline CP and its almost complete amorphisation. Thus, the amount of drug in the silica:drug ratio used in our study does not cause drug overloading, otherwise a thick CP layer would occur on the outer surface of the particle, leading to its crystallisation [12].

As shown in Figure 3b, no crystallinity was observed for bare MSN by XRD analyses, as expected, whereas a crystalline diffraction pattern was evidenced for the CP raw material. On the other hand, no peaks were observed for the MSN-CP-3-1 samples, matching the crystalline CP diffraction profile, although its DSC profile showed a trace endothermic peak. The detection limit of the XRD analysis is approximately 5%, which makes XRD less sensitive to traces of crystallinity than DSC [38,39]. One possible explanation for the disagreement in these results could be the very low degree of crystallinity in the MSN-CP-3-1 formulation, which is not possible to detect by XRD analysis. Therefore, from these results, we can suggest that most of the CP is loaded in the amorphous form into the pores of the MSN.

**Figure 4:**
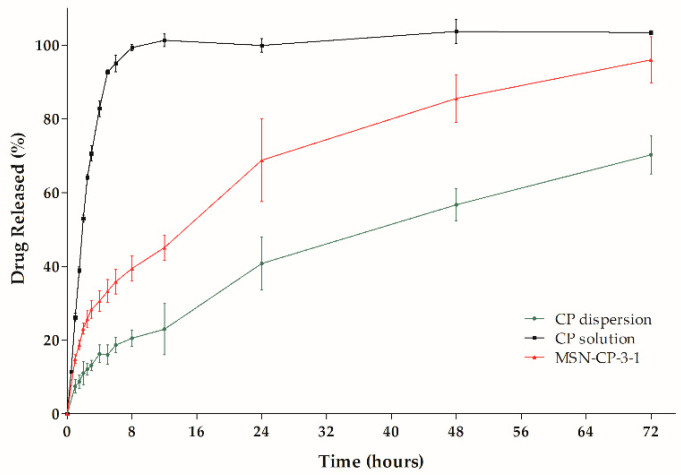
Clobetasol propionate (CP) in vitro release profile from: CP-loaded MSN (MSN-CP-3-1) and non-encapsulated CP (ethanolic solution and crystalline CP dispersion) (*n* = 3).

In the next step, the dissolution profiles of crystalline CP suspension, CP solution and CP from the MSN-CP-3-1 sample were evaluated using the dialysis bag method. Data are shown in Figure 4. CP was 100% dialysed from the ethanolic CP solution after 8 h, showing its fast diffusion through the dialysis bag. On the other hand, crystalline CP dispersed in an aqueous medium (as a suspension) had a poor release through the dialysis bag, due to its low solubility in the inner medium and consequently low dissolution rate.

These differences can be better understood by comparing the DE of the samples, whose values were calculated as 40.79 ± 4.75%, 65.70 ± 6.52% and 97.40 ± 1.81%, for the CP dispersion, MSN-CP-3-1 and ethanolic CP solution, respectively. A clear enhancement in the DE of the drug-loaded MSN (MSN-CP-3-1) (*p* ≤ 0.05) was observed compared to the crystalline CP suspension.

The observed differences in dissolution profiles of the samples are supported by the surface area, pore volume, DSC and X-ray analyses previously discussed. MSN-CP-3-1 had partially filled pores, weak CP endothermic peak intensity and no traces of crystalline CP in the X-ray pattern. Thus, CP is likely to be well dispersed in the larger surface area of the MSN and mostly loaded inside the MSN pores, favouring the drug dissolution process. This incorporation of the drug significantly changes the crystalline CP to a mainly non-crystalline state (almost complete amorphisation), enhancing its apparent solubility. Therefore, this formulation could be used in the development of 3D printing skin delivery films, as will be discussed in the next steps of this study.

### 3.2. Hydrogel Printing Inks

**Figure 5:**
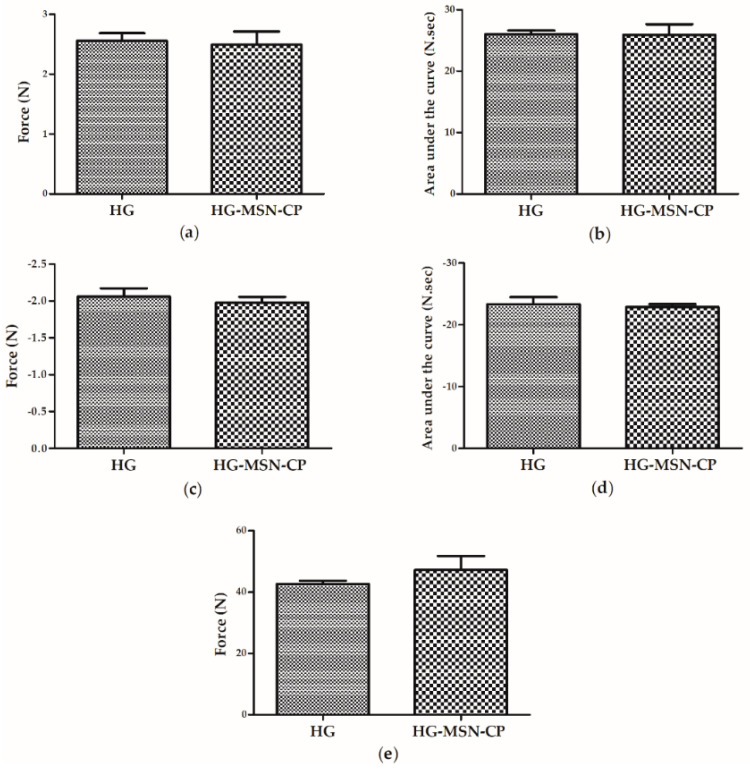
Texture properties of the hydrogel (*n* = 3) with or without CP-loaded MSN (HG and HG-MSN-CP), using the backward extrusion test (a–d) and the forward extrusion test (**e**). (**a**) Firmness (backward extrusion); (**b**) Consistency; (**c**) Cohesiveness; (**d**) Index of Viscosity; and (**e**) Firmness (forward extrusion). Data are expressed as mean ± SD. Student’s t-test was used to compare the samples. No differences between the samples were observed.

The printing inks were composed of pectin and CMC, as hydrogel forming polymers. Glycerine was added to the inks as a plasticiser and moisturiser. This composition was designed according to preliminary studies regarding their concentration in the blend, as well as their benefits for topical skin administration. CMC is a gel-forming agent, widely applied in wound dressing [40], whereas pectin is widely known for its bioadhesion properties [41,42]. After preparation of the hydrogel, MSN-CP was loaded in the printing ink by geometric dilution and under gentle manual homogenisation. Thus, two different inks were prepared and characterised, one containing the MSN-CP (HG-MSN-CP) and one not (HG). The rheological properties of these semisolid inks were evaluated before the 3D printing process, as they can affect the printability and the design fidelity. First, the backward and forward extrusion behaviours of the hydrogels were studied. The backward extrusion test (Figure 5a–d) gives an indication of the physical failure, consistency and cohesiveness parameters of the product, which are important aspects to be observed during the SSE 3D printing process. No difference (*p* > 0.05) was observed between the behaviour of the two formulations (HG-MSN-CP and HG), showing that the MSN-CP do not influence these properties. Recently, higher firmness, consistency, cohesiveness and index of viscosity values were reported for a hydrogel ink composed of CMC and glycerine only [20]. The difference in these properties compared to the hydrogel proposed here could be attributed to the lower concentration of CMC in the semisolid (5% versus 2.5%), as well as the presence of pectin in our hydrogel. The results can be translated to the printing behaviour and printing parameters of the two hydrogels: the hydrogel proposed by de Oliveira et al. (2022) [20] was able to print objects with a height of 2.5 mm, whereas films higher than 2 mm tended to collapse in our study. A similar behaviour was observed for the forward extrusion analysis (Figure 5e), where no difference in the firmness shown by the two inks was evidenced (HG-MSN-CP and HG), although the results were lower than the findings reported by Oliveira et al. (2022) [20] for an ink composed of CMC only.

**Figure 6:**
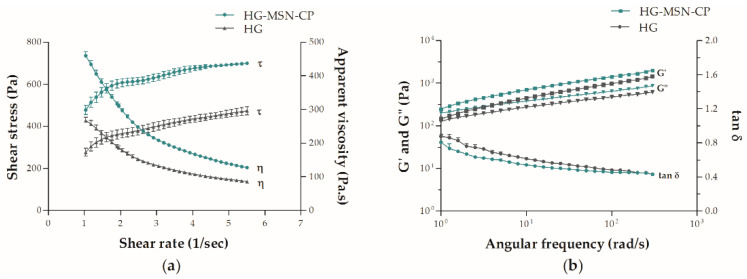
Rheological behaviour of the following printing inks: HG-MSN-CP and HG-CP. (**a**) Flow curves are shown as shear stress (τ) and apparent viscosity (η) versus shear rate, and (**b**) Oscillatory frequency test demonstrated as storage modulus (G’), loss modulus (G”) and tangent delta (tan δ) versus angular frequency. Values are presented as mean ± SD (*n* = 3).

For a deeper rheological understanding, analyses were carried out using a rheometer. Figure 6a shows the rheological behaviour of HG-MSN-CP and HG-CP, both demonstrating pseudoplastic characteristics (shear-thinning behaviour), which could be interpreted as a decrease of the viscosity by an increase of the shear rate. A shear-thinning behaviour of inks is usually desired in the 3D printing process, as it favours the flowability of the material during extrusion.

In addition, rheological analyses revealed a slightly higher complex viscosity for HG-MSN-CP (77 ± 2) than HG-CP (70 ± 1) at 1 Hz (*p* ≤ 0.05). However, the yield stress, which is the force that must be applied to the ink for it to begin to flow, was no different between the formulations (*p* > 0.05), which means that the same force was required for both inks to begin to flow. Besides, both hydrogels showed G’ higher than G’’ (tan < 1), demonstrating a predominant elastic behaviour (Figure 6b), which is typical of the gel-like state, thereby contributing to the recovery of their viscosity and shear retention after extrusion. In addition, G’ was higher (lower tan δ) for HG-MSN-CP than HG-CP at the same angular frequency, which could indicate that the presence of MSN-CP provides a better shape retention of the films after extrusion. Schmidt and co-workers (2022) also evaluated the influence of some texture properties of MSN in CMC hydrogels, whose results concur with our present findings, suggesting that MSN can play an important role in increasing the viscosity of 3D printing ink and should be further evaluated in future studies [21].

In general, for an efficient SSE 3D-printing process, the semisolid used as the printing ink should present three main characteristics: high viscosity during rest, a decrease of viscosity under the shear stress through the nozzle, and a fast recovery of its viscosity after deposition on the printing platform [43]. A low viscosity of the semisolid could make it easier to extrude through the nozzle, although a high apparent viscosity, as shown by the HG-MSN-CP, could be associated with a stronger mechanical integrity and shape retention, which are some of the desired properties of inks during an SSE process and may help the preparation of 3D-printed skin delivery films.

3.3. 3D Printing of Skin Drug Delivery Films

**Figure 7:**
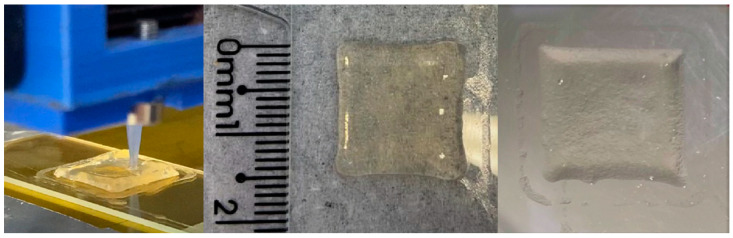
3D-printed film (sample F-HG). From left to right: 3D printing during extrusion; 3D-printed film before drying; 3D-printed film after drying.

Drug delivery films intended for cutaneous administration were 3D printed by SSE and left to dry at room temperature for 72 h. Their size, weight and thickness reduced visually during the drying step, due to the water loss (Figure 7). The final size of the films was 13.82 ± 0.63 mm × 13.98 ± 0.20 mm for F-HG-MSN-CP and 13.14 ± 0.51 mm × 13.10 ± 0.19 mm for F-HG, whereas their thickness was 0.29 ± 0.015 mm and 0.32 ± 0.059 mm, respectively. These dimensions afforded films with a final mean weight of 73.47 ± 6.28 mg and 69.90 ± 10.28 mg for F-HG-MSN-CP and F-HG, respectively. The presence of the MSN did not influence any of these properties (*p* > 0.05) or the visual aspect of the films.

Size and thickness are essential features to consider during the design and development of new skin delivery formulations. These properties impact directly on patient acceptance and, thereafter, on the treatment efficacy. One of the main advantages of using 3D printing in pharmaceutics is the personalisation of therapies, including the customisation of the size and thickness of dermatological formulations. Besides the size and thickness optimised in this study, the films were also 3D printed varying these parameters (15 mm × 15 mm × 1.5 mm, 15 mm × 15 mm × 2 mm, 5 mm × 5 mm × 1 mm), evidencing that this ink composed of CMC and pectin allows the personalisation of delivery systems (data not shown). Therefore, this approach could soon be translated into 3D printing drug delivery films of the specific size of the patient’s skin lesion and/or with a specific dose per unit.

Regarding the drug content, the expected amount of drug per film was calculated as 33.8 mg/unit, according to the final physical dimensions. The films were produced with a mean experimental drug content of 35.52 ± 2.35 mg per film, equivalent to 105.15% of the expected dose. This dose represents approximately 0.48 ± 0.032 mg/g per film (0.048% *w/w* of drug loading), like the commercially available concentration of the CP formulation for skin administration (0.05% *w/w*). In addition, the drug dose was reproducible, considering a sequence of three printed films, which had a low relative standard deviation (6.61%). These data suggest that CP-loaded silica particles could be well dispersed in the ink, affording a good drug homogeneity among films printed in a sequence. Along with the potential of dose customisation of these 3D-printed skin delivery systems, this process could assure a better control of the applied dose compared to the administration of semisolid dermatological formulations, such as creams, lotions and gels, where it is often difficult to control the exact applied dose.

**Figure 8:**
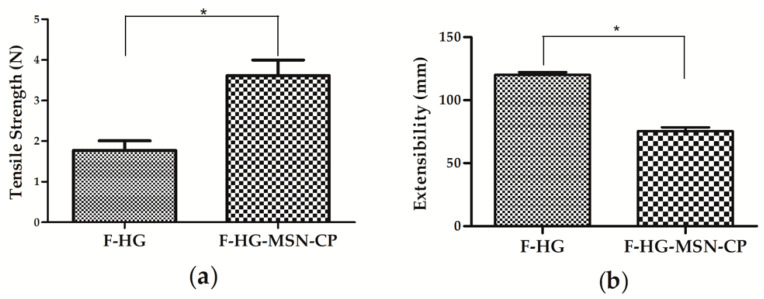
Tensile strength (**a**) and extensibility (**b**) of 3D-printed films: F-HG and F-HG-MSN-CP (*n* = 3, mean ± SD). Student’s t-test was used to compare the samples. * *p* ≤ 0.05.

Furthermore, it is important to study the mechanical properties of skin delivery films, as they must simultaneously be resistant, elastic and easy to handle, store and transport. The tensile strength of the 3D-printed films (F-HG-MSN-CP and F-HG) are depicted in Figure 8. A higher force was needed to break the F-HG-MSN-CP (3.613 ± 0.38 *n*) compared to the F-HG (1.773 ± 0.24 *n*), meaning that the presence of MSN increased the mechanical strength of the CMC and pectin film. On the other hand, the extensibility of F-HG-MSN-CP (75.39 ± 3.01 mm) was lower than F-HG (120 ± 2.15 mm), which means that the latter stretches to a relatively longer distance before breaking. This lower extensibility for F-HG-MSN-CP could be related to its higher solid content, which has been previously reported as a factor responsible for lowering the flexibility of polymeric films [44]. An increase of mechanical strength and decrease of the extensibility of 3D-printed CMC films was also reported after the addition of mesoporous silica (SBA-15) to the printing ink [21]. These data are in agreement with previous studies relating the concentration of pectin in polymeric films to their tensile strength properties [45].

**Figure 9:**
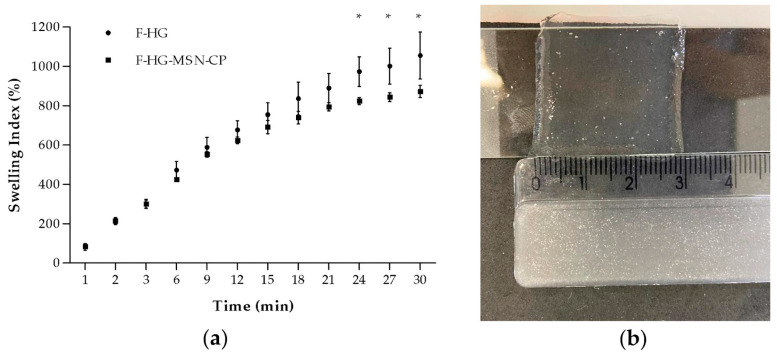
Swelling index profiles of 3D-printed films (**a**): F-HG and F-HG-MSN-CP (*n* = 3, mean ± SD). Appearance of the F-HG-MSN-CP after 30 min (**b**). Two-way ANOVA and Bonferroni post hoc test. * *p* ≤ 0.05.

Another critical feature of films for skin administration is their swelling index profile, which can be related to their bioadhesion properties. Figure 9 (left) shows the swelling index profile of the films with or without MSN-CP. No statistical difference was observed between the two profiles during the first 20 min. However, after 24 min, the films showed different swelling indices (*p* ≤ 0.05), indicating that the MSN has an influence on the swelling properties of the 3D-printed films, even if added in a small amount (approximately 0.4 mg of MSN per 1 g of hydrogel). The F-HG-MSN-CP had a smaller swelling index value compared to F-HG, which can be attributed to the presence of the mesoporous silica. However, after 30 min, films prepared without MSN-CP (F-HG) started to lose their integrity, making them difficult to handle and weigh after this time. This loss of integrity was not observed for F-HG-MSN-CP, which were still totally intact after 30 min (swelling index of 873 ± 31%). Figure 9b shows the image of F-HG-MSN-CP after 30 min, with the size roughly doubled. This important characteristic brings an advantage for the use of mesoporous silica, as the films were able to absorb liquid and maintain their integrity for a longer period. Schmidt and co-authors (2022) recently reported a lower swelling index after 30 min for buccal films produced by SSE 3D printing from an ink composed of CMC and glycerine [21]. The higher swelling index reported here can be attributed to the presence of pectin, which drastically improved the ability of the films to absorb water.

**Figure 10:**
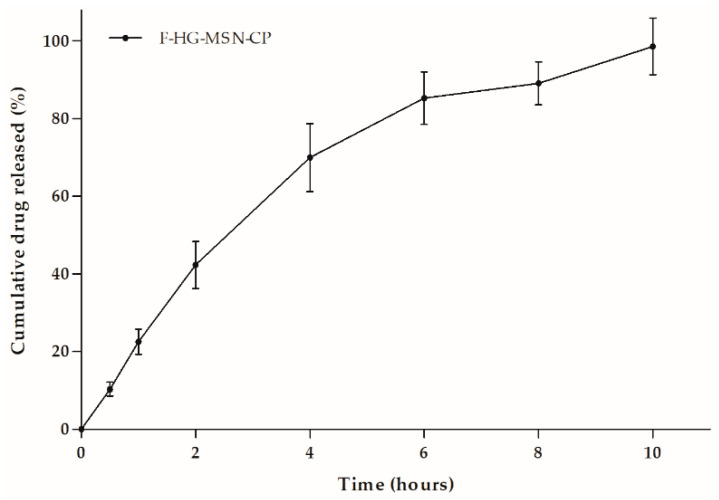
Cumulative drug release (%) of 3D-printed F-HG-MSN-CP, evaluated using the Franz diffusion method.

To evaluate whether CP release from the F-HG-MSN-CP would be complete in a reasonable time, the in vitro release profile was performed using Franz diffusion cells and synthetic dialysis cellulose as the membrane, to avoid transfer of the CP still loaded in the MSN to the receptor medium. The release profile of 3D-printed F-HG-MSN-CP is displayed in Figure 10, which shows the cumulative CP release over time. An initial fast release was observed in the first 2 h (~40%), followed by a slower release phase, reaching complete drug release (100%) after 10 h. The loading of the lipophilic drug (CP) in MSN was a strategy to its formulation as hydrophilic films composed of CMC and pectin, and these data reveal the suitability of their design as skin delivery systems, with drug release in less than 12 h.

**Figure 11:**
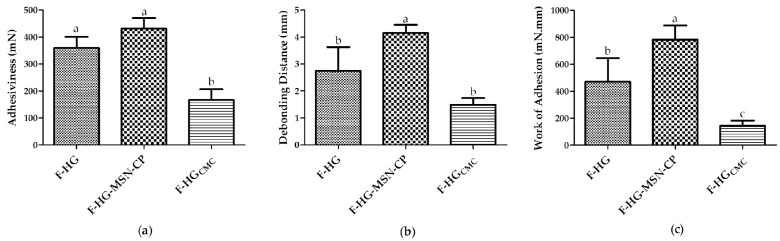
Bioadhesion measurements: (**a**) adhesiveness (mN), (**b**) debonding distance (mm) and (**c**) work of adhesion (mN·mm) of the 3D-printed films (F-HG, F-HG-MSN-CP and F-HGCMC). Data represent mean ± SD, *n* = 3. One-way ANOVA and Tukey post hoc test. Means with different letters denote statistical difference (*p* ≤ 0.05).

Finally, the bioadhesion properties of the 3D-printed films were evaluated using pig skin as the biological membrane model, due to its similarities to human skin. Bioadhesion can be defined as the attachment of a formulation to a biological tissue. To evaluate the bioadhesion properties of the 3D-printed films, an in vitro study was designed to compare films composed of CMC and pectin with or without MSN-CP (F-HG-MSN-CP and F-HG, respectively) and films composed only of CMC (F-HGCMC). Studies were carried out to understand the influence of the MSN and/or pectin on the in vitro bioadhesion properties of the 3D-printed films prepared according to the platform proposed in our study. As shown in Figure 11, the presence of MSN-CP showed no influence on the films’ adhesiveness (F-HG-MSN-CP × F-HG, *p* > 0.05), whereas both films showed a better adhesiveness than the film composed of CMC only (Figure 11a).

Moreover, all the formulations showed a different work of adhesion (F-HG-MSN-CP > F-HG > F-HGCMC) (Figure 11c). These results mean that the work and the force required to remove them from the skin are different. The work necessary to remove the films containing pectin was higher (470 ± 175 mN·mm) than that required to remove the film composed of CMC (144 ± 38 mN·mm), while the presence of MSN also improves the work of adhesion (782 ± 105 mN·mm), reflecting a better skin adhesion (bioadhesion). These data agree with our recent report, where the positive effect of another kind of MSN (SBA-15) was observed on the mucoadhesion behaviour of 3D-printed buccal films composed of CMC only [21]. Although the substrates in these studies were different (porcine ear skin and mucin disc models), this reinforces the potential of MSN to improve the bioadhesion (skin or mucosa) of 3D-printed films. Furthermore, these data are corroborated by the debonding distance, which represents the distance travelled by the probe for complete displacement from the skin. F-HG-MSN-CP showed the highest value (Figure 11b), reflecting the improved skin adhesion behaviour due to the presence of pectin in the formulation, which is a polymer widely known to add adhesion properties to different kind of formulations [46,47]. Consequently, one of our initial hypotheses was confirmed: the presence of pectin in the hydrogels improved the skin adhesion of 3D-printed films intended for topical administration, together with a slight effect from the MSN on this adhesion property. This effect of the MSN on the skin adhesion should be further investigated in future studies.

## 4. Conclusions

In this study, a novel 3D-printed drug-loaded hydrophilic skin delivery film was developed from a printing ink composed of CMC (5% *w/v*) and pectin (5% *w/v*), as polymers. The loading of CP in the MSN noticeably reduced its degree of crystallinity, resulting in an increase of the intrinsic aqueous solubility, making possible the redispersion of an hydrophobic drug (CP) in a novel hydrophilic blend polymer. This nanomaterial was easily redispersed in the printing ink and provided a better rheological behaviour, related to the longer shape retention of the films after SSE. The 3D-printed films had a homogeneous interindividual drug dose (0.048% *w/w*), enhanced mechanical properties due to the presence of MSN, and complete drug release in 10 h. Moreover, they showed good swelling behaviour (873 ± 31% in 30 min) and outstanding skin bioadhesion behaviour (work of adhesion of 782 ± 105 mN·mm), mainly due to the presence of pectin in the ink. Therefore, this study represents a proof of concept regarding the preparation of bioadhesive skin delivery films by 3D printing, combining the use of advanced nanomaterials and additive manufacturing. Further studies will be designed to evaluate the influence of the MSN in these films on the skin drug permeation and penetration behaviour.

## Figures and Tables

**Table 1:** Surface area and pore volume values of MSN (MCM-41) and MSN-CP-3-1.

Sample	BET Surface Area (m^2^ g^−1^)	Pore Volume (cm^3^ g^−1^)
MSN	899	0.731
MSN-CP-3-1	291	0.198

## Data Availability

The datasets generated during and/or analysed during the current study are available from the corresponding author on reasonable request.

## Supplementary Materials

The following supporting information can be downloaded at: https://www.mdpi.com/article/10.3390/pharmaceutics15010020/s1, Figure S1: Scanning electron microscopy (SEM) image of bare MSN (MCM-41) at different magnifications: (**a**) 10,000× and (**b**) 20,000×. Figure S2: X-ray Powder Diffraction (XRD) analysis of MSN in the range of 2θ = 1 to 10°
