# Supplementary material for: Bioadhesive 3D-Printed Skin Drug Delivery Polymeric Films: From the Drug Loading in Mesoporous Silica to the Manufacturing Process
Source: Pharmaceutics. 2022 Dec 21;15(1):20. doi: 10.3390/pharmaceutics15010020 (PMC9861290; doi:10.3390/pharmaceutics15010020)
Supplement: Supplementary file 1 [file pharmaceutics-15-00020-s001.zip › pharmaceutics-2073210-supplementary.pdf]

Supplementary material for

Bioadhesive 3D printed skin drug delivery polymeric films: from the drug loading in mesoporous silica to the manufacturing process

Rafaela Santos de Oliveira[1,4a], Nadine Lysyk Funk[1,4a], Juliana dos Santos[1,4], Thayse Viana de Oliveira[1,4], Edilene Gadelha de Oliveira[1], Cesar Liberato Petzhold[2], Tania Maria Haas Costa[2], Edilson Valmir Benvenutti[2], Monique Deon[3,4], Ruy Carlos Ruver Beck[1,4]*

[1] Programa de Pós-Graduação em Ciências Farmacêuticas, Faculdade de Farmácia, Universidade Federal do
Rio Grande do Sul, Porto Alegre 90610-900, Brazil; rafaelasantosooliveira@gmail.com (R.S.O.); nadine.lysyk@ufrgs.br (N.L.F.); santos.juliana@ufrgs.br (J.d.S.); thaysevfarma@gmail.com (T.V.d.O.); edileneego@yahoo.com.br (E.G.d.O.)

[2] Instituto de Química, Universidade Federal do Rio Grande do Sul, Porto Alegre 90650-001, Brazil; petzhold@iq.ufrgs.br (C.L.P); taniaha@iq.ufrgs.br; benvenutti@ufrgs.br (E.V.B.)

[3] Departamento de Farmacociências, Universidade Federal de Ciências da Saúde de Porto Alegre, Porto Alegre 90050-170, Brazil; monique.deon@ufcspa.edu.br (M.D)

[4] Laboratório de Nanocarreadores e Impressão 3D em Tecnologia Farmacêutica (Nano3D), Faculdade de Farmácia, Universidade Federal do Rio Grande do Sul, Porto Alegre 90610-000, Brazil; ruy.beck@ufrgs.br

[a] These authors contributed equally

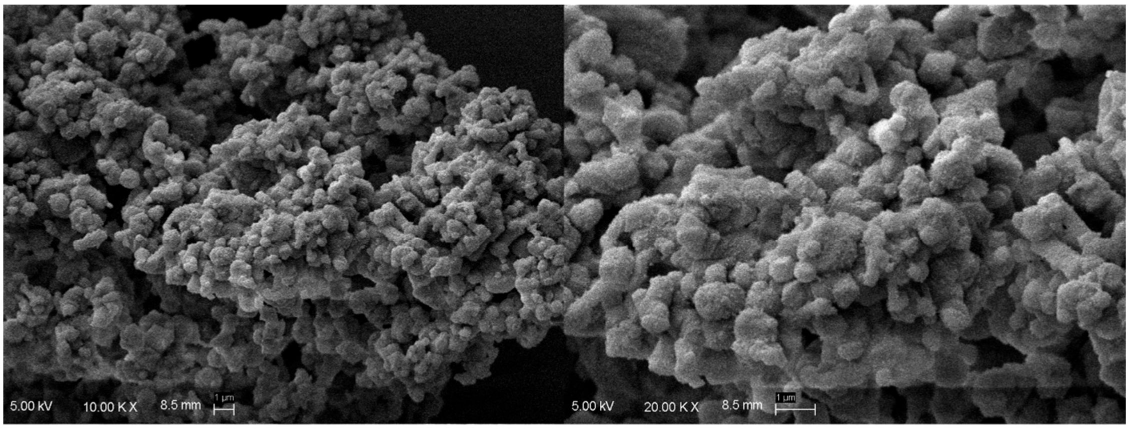

(**a**) (**b**)

S1. Scanning electron microscopy (SEM) image of bare MSN (MCM-41) at different magnifications: (**a**) 10,000x and (**b**) 20,000x.

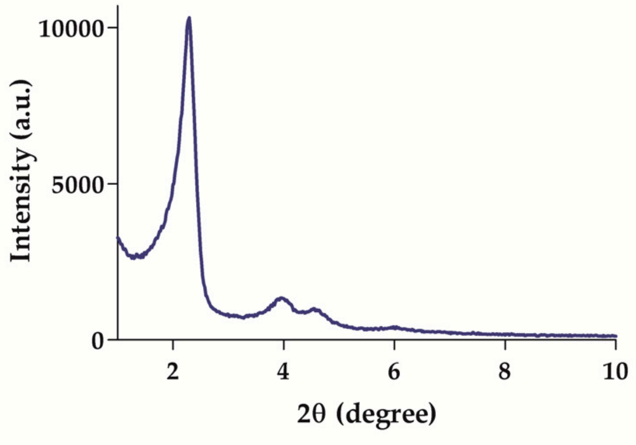

S2. X-ray Powder Diffraction (XRD) analysis of MSN in the range of 2θ = 1 to 10º.
